# Supplementary material for: An elastic element in the protocadherin-15 tip link of the inner ear
Source: Nat Commun. 2016 Nov 18;7:13458. doi: 10.1038/ncomms13458 (PMC5120219; doi:10.1038/ncomms13458)
Supplement: Supplementary Information — Supplementary Figures 1-17, Supplementary Tables 1-3, Supplementary Discussion and Supplementary References [file ncomms13458-s1.pdf]

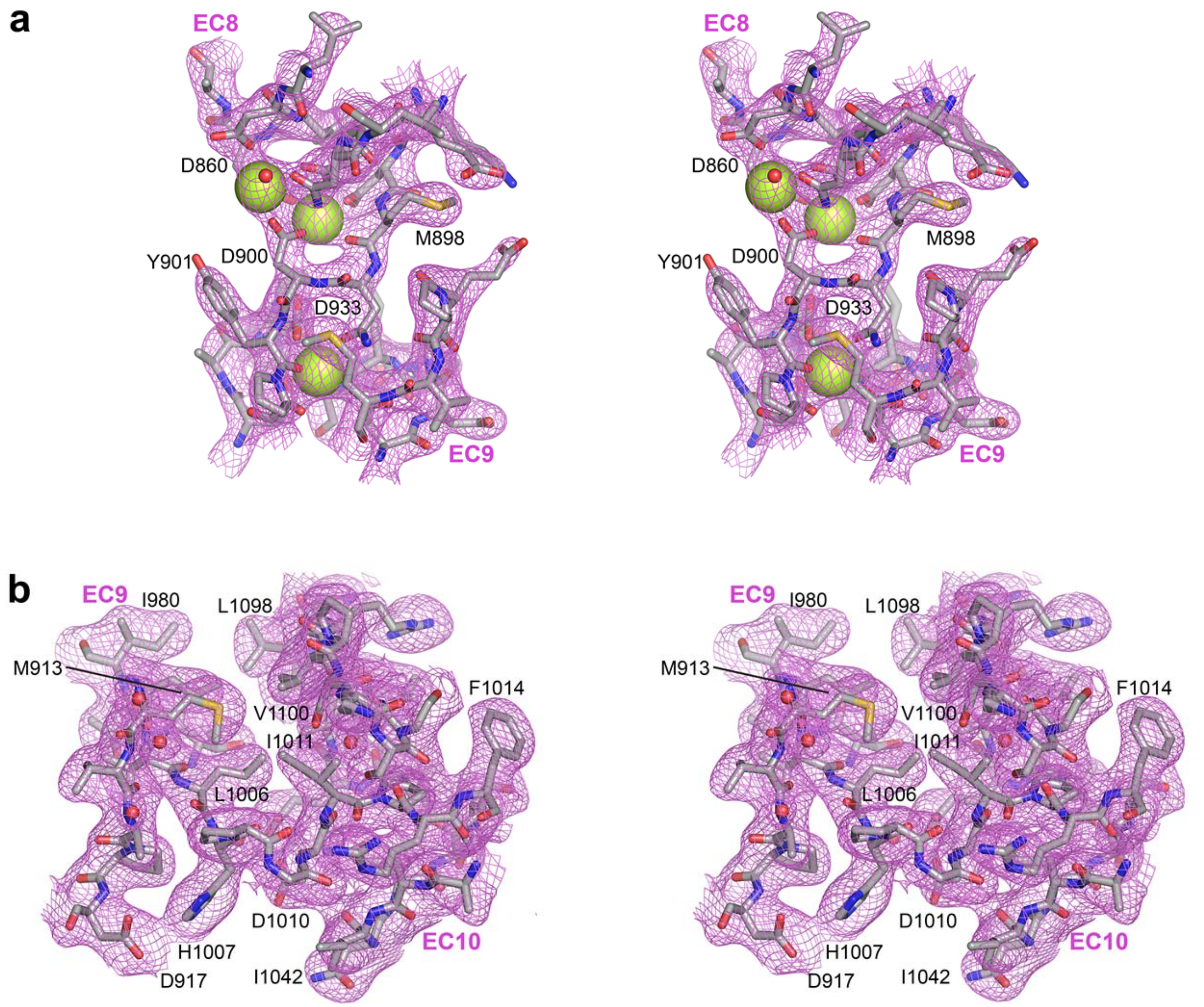

Supplementary Figure 1. Electron density map. Stereo view of the 2Fo-Fc electron density map (purple mesh) from the PCDH15 EC8-10 structure. **(a)** Detail of the density for the EC8-9 linker contoured at 2.0 σ. **(b)** Detail of the EC9-10 interface contoured at 0.7 σ.

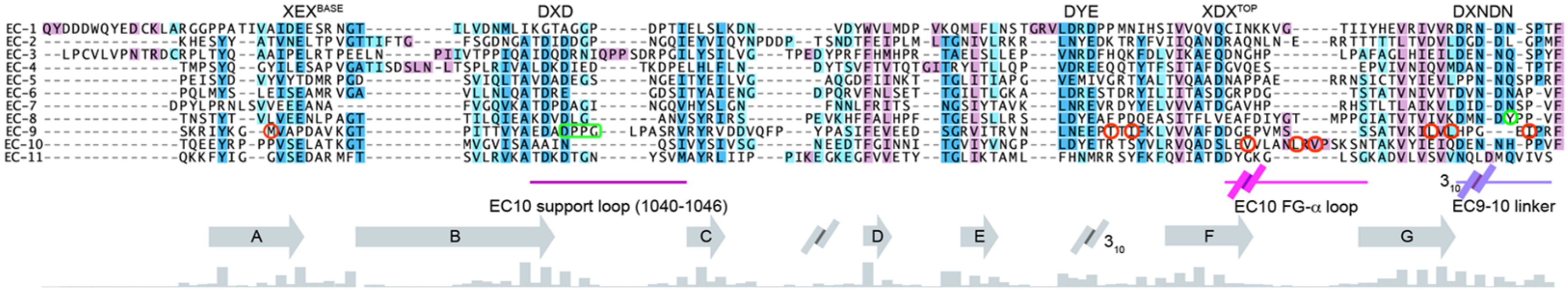

Supplementary Figure 2. Sequence alignment of human PCDH15 EC repeats. All 11 EC repeats are aligned to each other (EC1 to EC11). Conserved calcium-binding motifs are labeled as in[1]. Residues that form the hydrophobic core of the EC9-10 interface are highlighted with red circles. Residues unique to the EC8-9 linker are highlighted with green circles. Secondary structure of PCDH15 EC8 and sequence conservation are shown in gray below the alignment. Sequence features unique to the EC9-10 interface are labeled and highlighted with colored solid lines.

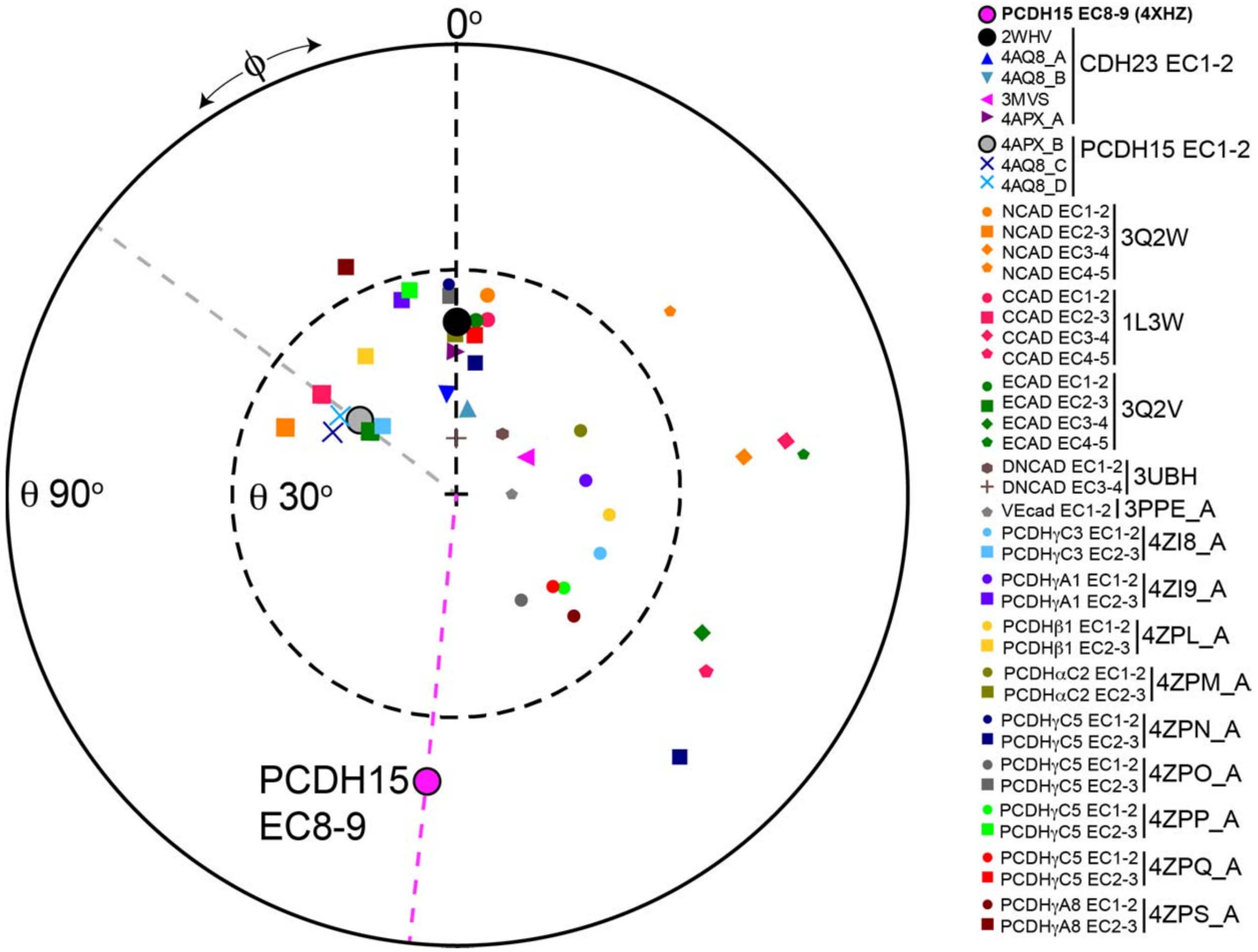

Supplementary Figure 3. Orientation of EC repeats in available cadherin structures. Orientation of tandem EC repeats for listed cadherins and PCDH15 EC8-9 (shown as in Fig. 2e). The N-terminal EC repeat for labeled structures was used as reference and aligned to the *z*-axis. CDH23 EC1-2 was used to define ϕ = 0°. The azimuthal angle (ϕ) observed for PCDH15 EC8-9 is unique.

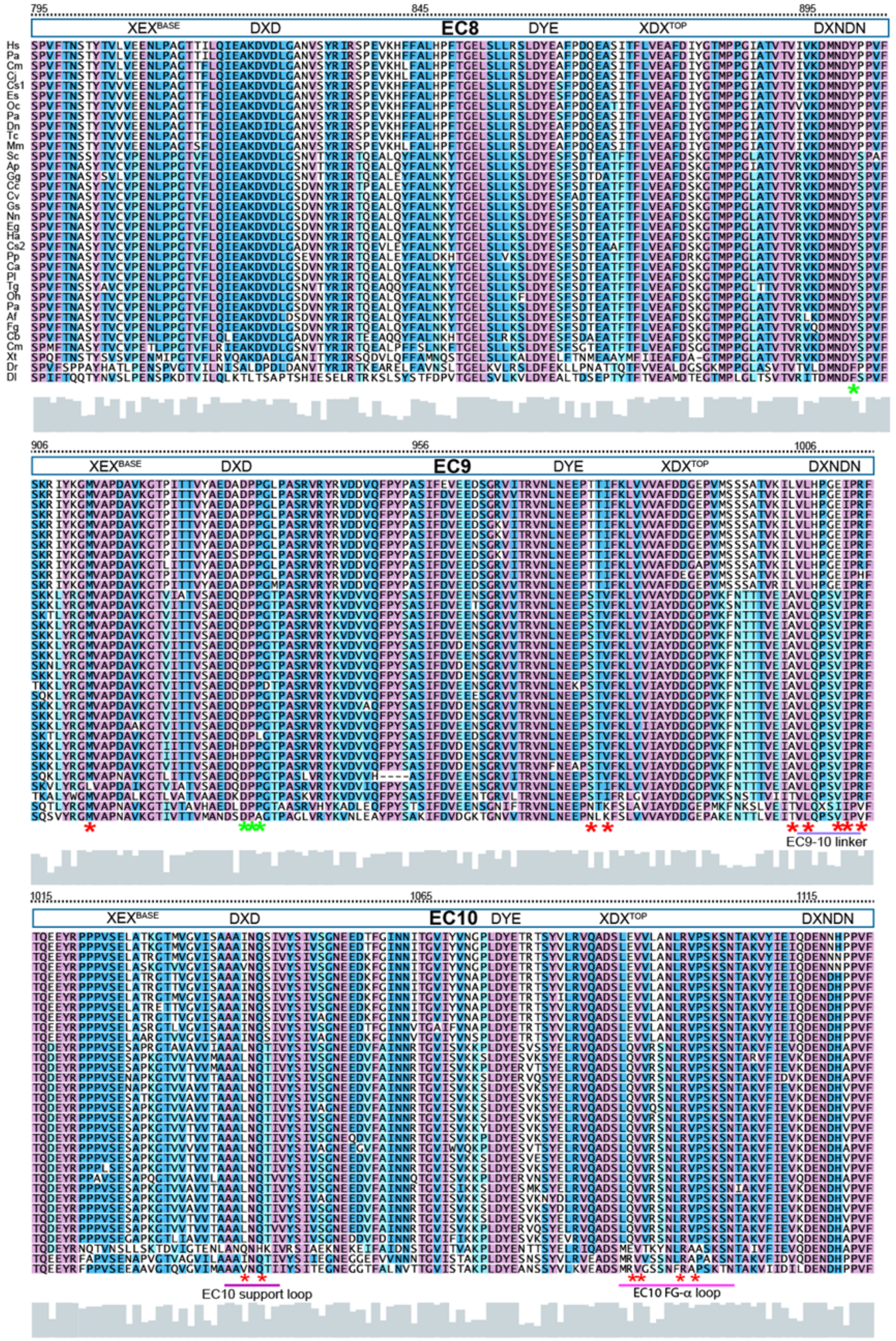

Supplementary Figure 4. Conservation of PCDH15 EC8-10. Alignment of PCDH15 sequences corresponding to EC8 (top), EC9 (middle) and EC10 (bottom) for *Homo sapiens (Hs), Papio Anubis (Pa), Callicebus moloch (Cm), Callithrix jacchus (Cj), Cynopterus sphinx (Cs1), Eonycteris spelaea (Es), Oryctolagus cuniculus (Oc), Pteropus alecto (Pa), Dasypus novemcinctus (Dn), Tupaia chinensis (Tc), Mus musculus (Mm), Struthio camelus (Sc), Anas platyrhynchos (Ap), Gallus gallus (gg), Cuculus canorus (Cc), Charadrius vociferus (Cv), Gavia stellata (Gs), Nipponia Nippon (Nn), Egretta garzetta (Eg), Haliaeetus albicilla (Ha), Colius striatus (Cs2), Picoides pubescens (Pp), Cathartes aura (Ca), Phaethon lepturus (Pl), Tinamus guttatus (Tg), Opisthocomus hoazin (Oh), Pygoscelis adeliae (Pa), Aptenodytes forsteri (Af), Fulmarus glacialis (Fg), Corvus brachyrhynchos (Cb), Chelonia mydas (Cm), Xenopus tropicalis (Xt), Danio rerio (Dr), Dicentrarchus labrax (Dl)* (Uniprot Ref. seq.: Q96QU1, B1MTB2, B0CMA1, G3GAY7, G3GAY4, B7NZ90, L5KEI6, C3PT11, L9L351, Q99PJ1, A0A093H1I5, R0LBC5, Q0ZM14, A0A091GGF8, A0A099ZZ55, A0A093FY27, A0A091V5S4, A0A091J1Q2, A0A091NT34, A0A091KZS7, A0A093GBT0, A0A091LI05, A0A091TNA5, A0A099Z4Y6, A0A091W8V8, A0A093PA17, A0A087QU20, A0A093IN62, A0A091FAE4, M7APD2, F6SAL8, Q5ICW6, E6ZH93). Labels as in Supplementary Fig. 2

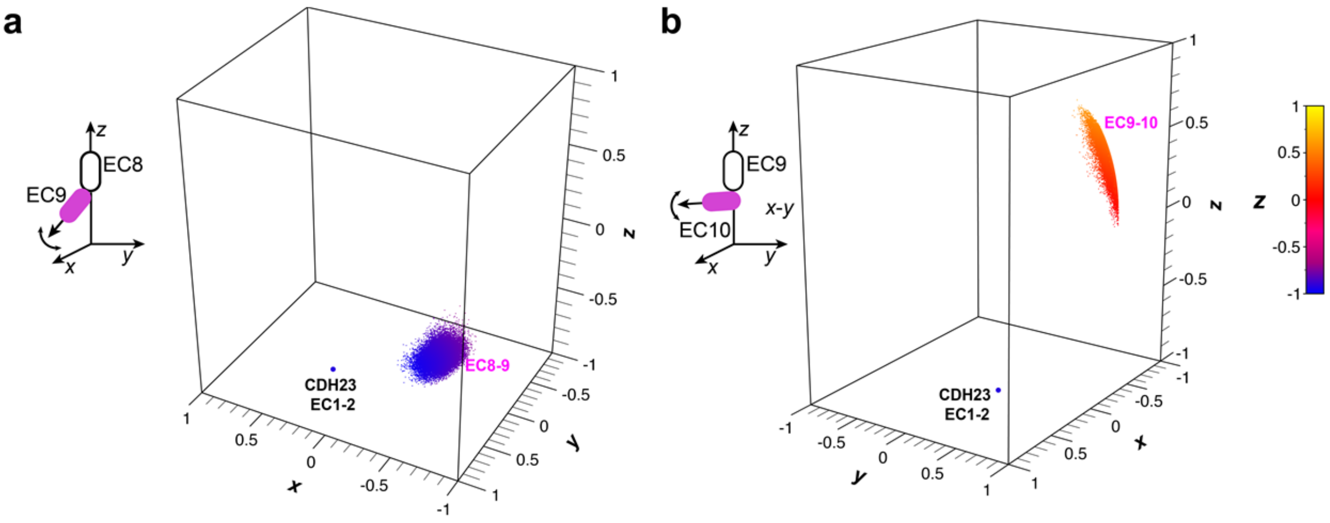

Supplementary Figure 5. Orientation dynamics of simulated PCDH15 EC repeats. Conformational freedom of **(a)** EC9 with respect to EC8 and of **(b)** EC10 with respect to EC9 are shown in 3D. Repeats EC8 and EC9 were aligned to the *z* axis and used as reference in each case. Dots represent the location of the tip of the principal axis for EC9 and EC10 throughout the trajectory. Color denotes position along *z*. Data taken from equilibrium simulation S1c. The orientation of CDH23 EC2 with respect to EC1 is included and labeled.

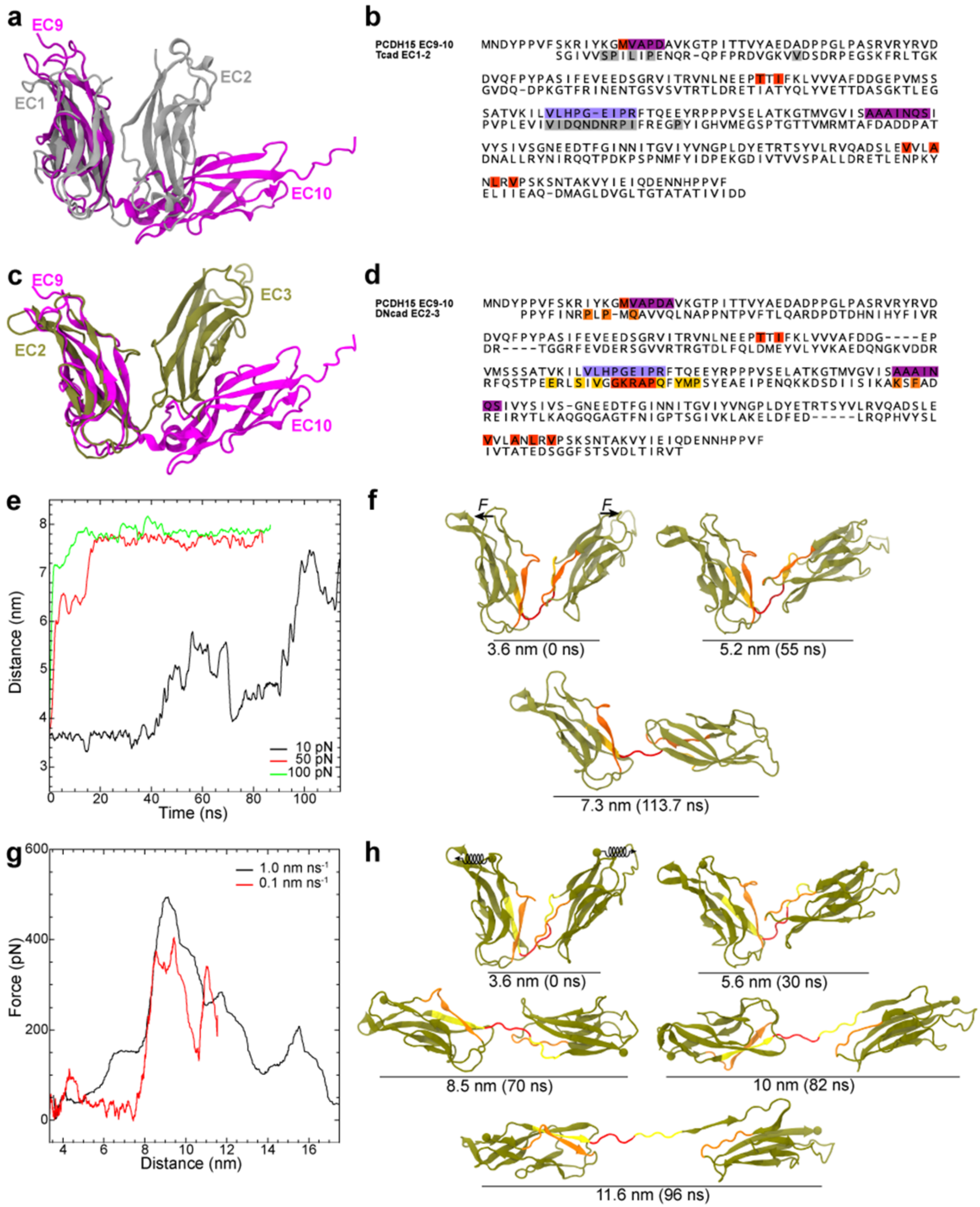

Supplementary Figure 6. Bent cadherin structures and SMD simulations of DNcad EC2-3. (**a**) Structural alignment of PCDH15 EC9 (4XHZ, magenta) and apo-Tcad EC1 (3K5R[2], gray). (**b**) Sequence alignment of PCDH15 EC9-10 and Tcad EC1-2. Colored boxes highlight structural features for PCDH15 EC8-10 as in Figs. 3, 6 and 7 and for Tcad gray boxes highlight residues that form the EC1-2 interface. (**c**) Structural alignment of PCDH15 EC9 (4XHZ, magenta) and DNcad EC2 (3UBG[3], olive). (**d**) Sequence alignment of PCDH15 EC9-10 and DNcad EC2-3. Colored boxes highlight structural features for PCDH15 EC8-10 as in Figs. 3, 6 and 7 and for DNcad EC2-3 as in panels f and h. (**e**) End-to-end distance vs. time (1 ns running average) for constant-force SMD simulations of DNcad EC2-3 using: 10 pN (S11a, black), 50 pN (S11b, red), and 100 pN (S11c, green). (**f**) Snapshots taken from the 10 pN simulation of DNcad EC2-3. (**g**) Force applied to N-terminus vs. end-to-end distance (1 ns running average) for constant-velocity stretching of DNcad EC2-3 at 1 (S12a, black) and 0.1 nm ns^{-1} (S12b, red). (**h**) Snapshots taken from the 0.1 nm ns^{-1} simulation.

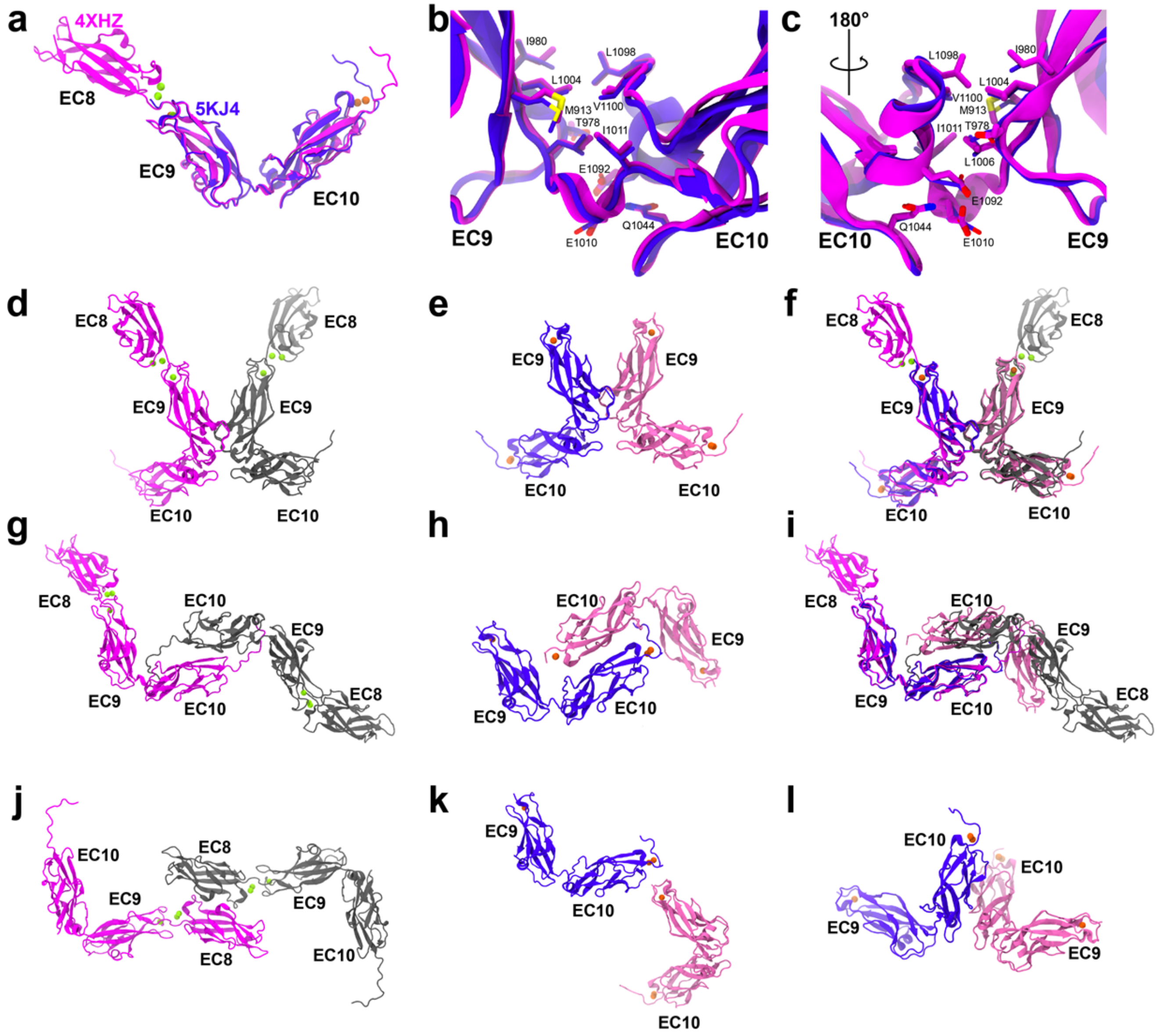

Supplementary Figure 7. Crystallographic models and contacts for PCDH15 EC8-10 and mmPcdh15 EC9-10. (a) Superposition of the EC8-10 and EC9-10 structures (PDB codes 4XHZ [protein as magenta ribbons and calcium ions as green spheres] and 5KJ4 [protein as violet ribbons and calcium ions as orange spheres]). **(b&c)** Detail of the EC9-10 interface in the structures shown in a. The protein backbone is shown as ribbons and relevant residues are shown as sticks. **(d,g,j)** Crystallographic contacts observed in 4XHZ [protein in magenta and gray; calcium ions as green spheres]. **(e,h,k,l)** Crystallographic contacts observed in 5KJ4 [protein in violet and pink.; calcium ions as orange spheres]. **(f)** Superposition of d and e. **(i)** Superposition of g and h.

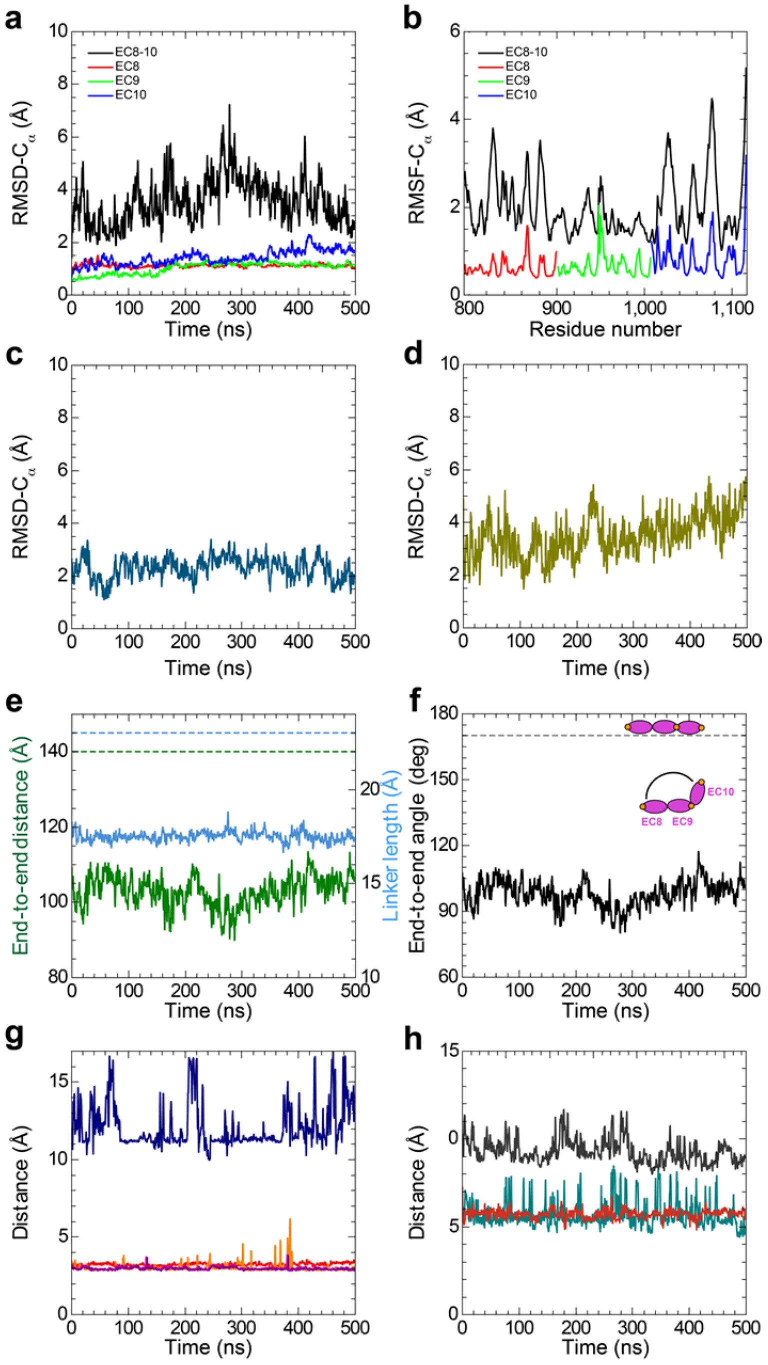

Supplementary Figure 8. Equilibrium properties of PCDH15 EC8-10 from simulation S1c. (**a**) RMSD-C_α and (**b**) RMSF-C_α for the complete structure (black) and for EC8 (red), EC9 (green), and EC10 (blue). RMSF-C_α computed for the complete structure (black) is artificially high due to inter-repeat motions. (**c**) RMSD-C_α of the EC9-10 3_{10} helix. (**d**) RMSD-C_α of the EC10 support loop. Crystal structure coordinates were used as reference in all cases. (**e**) End-to-end distance (Ser 975:C_α – Glu 1116:C_α; dark green, left scale) and EC9-10 linker length (Val 1005:C_α – Arg 1013:C_α; light blue, right scale). Dark green and light blue dashed lines show values for a straight conformation. (**f**) End-to-end angle of PCDH15 EC8-10 defined as the angle formed by atoms Ser 795:C_α – Val 1005:C_α – Glu 1116:C_α. Gray dashed line shows the angle for a straight conformation. (**g&h**) Interatomic distances for His 1007:O – Glu 1010:N (red), Gly 1009:O – Ile 1042:N (orange), Arg 1013:O – Ala 1040:N (magenta), Arg 1013:N – Ala 1040:O (purple), Glu 1010:C_γ – Arg 1013:C_ζ (blue), and Met 913:S_δ – Val 1100:C_β (teal), Leu 1004:C_γ – Leu 1098:C_γ (maroon) and Leu 1004:C_δ – Ala 1096:C_β (dark gray). A 1 ns running average is shown in all cases. Data shown in all panels taken from simulation S1c.

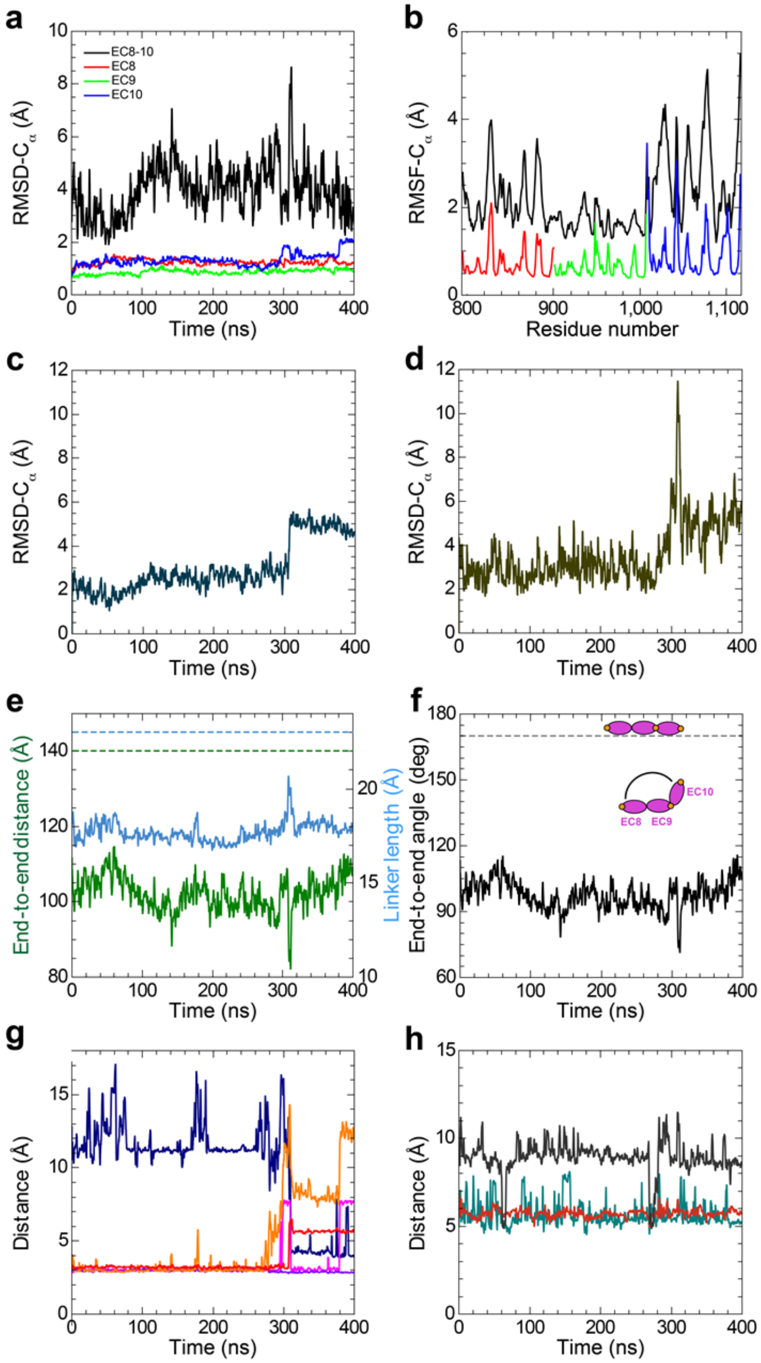

Supplementary Figure 9. Equilibrium properties of PCDH15 EC8-10 from simulation S1b. (**a**) RMSD-C_α and (**b**) RMSF-C_α for the complete structure (black) and for EC8 (red), EC9 (green), and EC10 (blue). RMSF-C_α computed for the complete structure (black) is artificially high due to inter-repeat motions. (**c**) RMSD-C_α of the EC9-10 3_{10} helix. (**d**) RMSD-C_α of the EC10 support loop. Crystal structure coordinates were used as reference in all cases. (**e**) End-to-end distance (Ser 975:C_α – Glu 1116:C_α; dark green, left scale) and EC9-10 linker length (Val 1005:C_α – Arg 1013:C_α; light blue, right scale). Dark green and light blue dashed lines show values for a straight conformation. (**f**) End-to-end angle of PCDH15 EC8-10 defined as the angle formed by atoms Ser 795:C_α – Val 1005:C_α – Glu 1116:C_α. Gray dashed line shows the angle for a straight conformation. (**g&h**) Interatomic distances for His 1007:O – Glu 1010:N (red), Gly 1009:O – Ile 1042:N (orange), Arg 1013:O – Ala 1040:N (magenta), Arg 1013:N – Ala 1040:O (purple), Glu 1010:C_γ – Arg 1013:C_ζ (blue), and Met 913:S_δ – Val 1100:C_β (teal), Leu 1004:C_γ – Leu 1098:C_γ (maroon) and Leu 1004:C_δ – Ala 1096:C_β (dark gray). A 1 ns running average is shown in all cases. Data shown in all panels taken from simulation S1b.

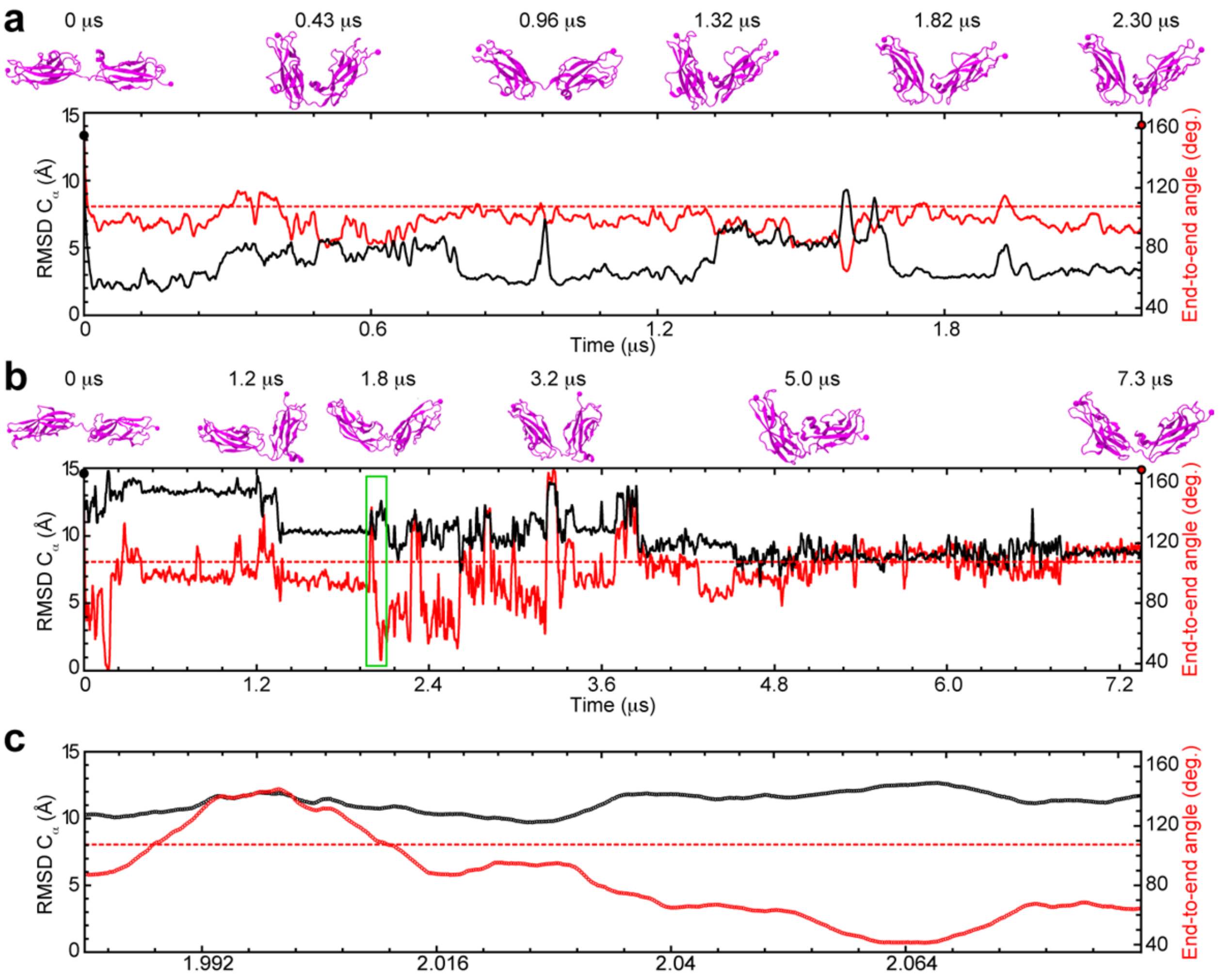

Supplementary Figure 10. Long timescale relaxation of PCDH15 EC9-10 stretched states. RMSD-C$_\alpha$ (**black**) and end-to-end angle (Asn 899:C$_\alpha$ – Val 1005:C$_\alpha$ – Glu 1116:C$_\alpha$; **red**) from "relaxation" equilibrium simulations of PCDH15 EC9-10 started from (**a**) S5b-75ns (S15a-b) and (**b**) S5b-76ns (S16a-b). (**c**) Detail from panel b (green box). Black and red circles indicate the starting values for RMSD-C$_\alpha$ and end-to-end angle respectively. Dashed red line indicates angle for crystal conformation. Snapshots above each panel depict representative conformations sampled during these simulations.

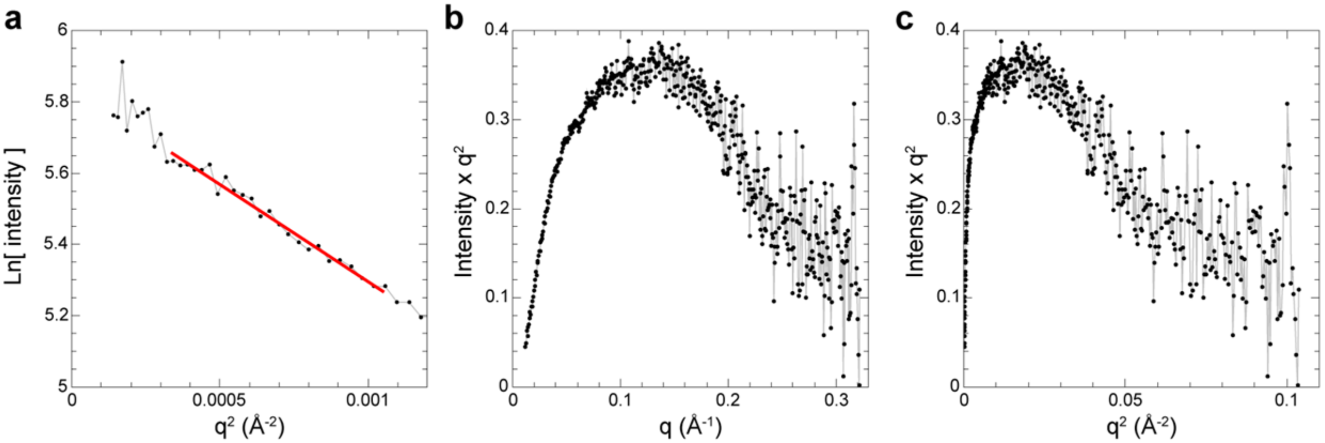

Supplementary Figure 11. Analysis of PCDH15 EC8-10 SAXS data. (**a**) Guinier plot of the low *q* region of the X-ray scattering. Red line shows the linear fit (qR_g range 0.9 – 1.3) from which the gradient of the slope ($-R_g^2/3$) is used to estimate R_g. (**b**) Kratky plot. (**c**) Kratky-Debye plot. The overall parabolic shape in both plots indicates that the protein is flexible but folded in solution[4].

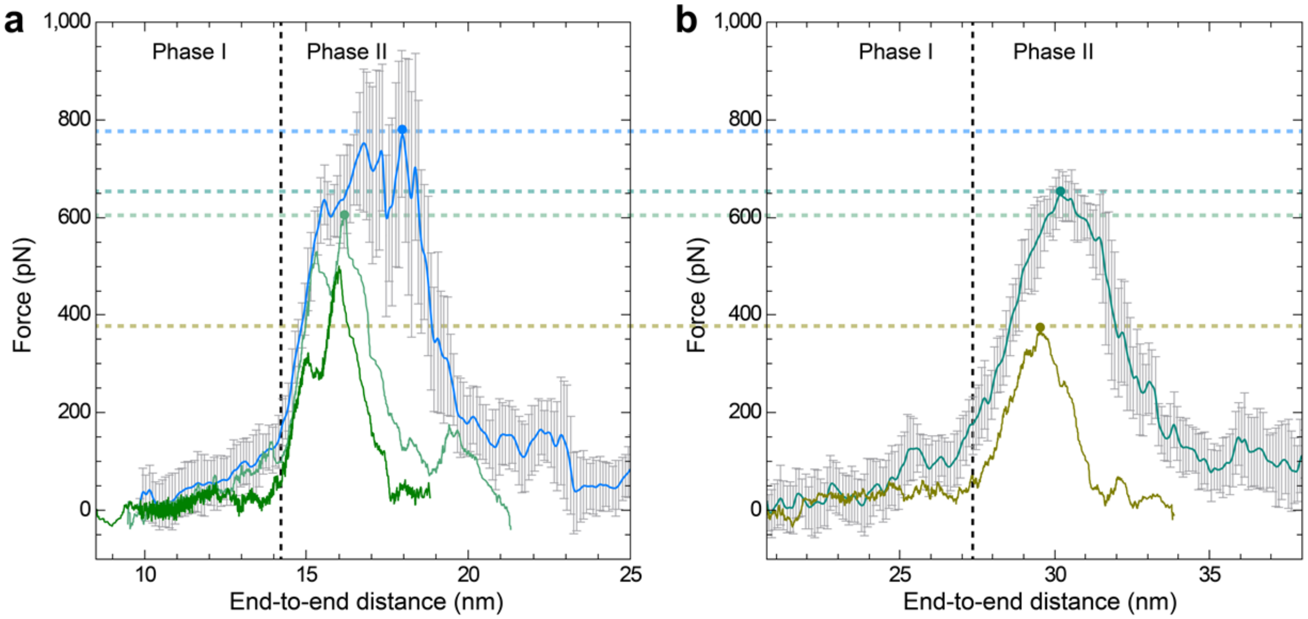

Supplementary Figure 12. Analysis of unfolding and unbinding forces in simulations. (a) Force applied to N-terminus vs. end-to-end distance for constant velocity stretching of PCDH15 EC8-10 at 1 nm ns^{-1} (average of S5a1-4, light blue), 0.1 nm ns^{-1} (S5b, light green) and 0.02 nm ns^{-1} (S5e, dark green). **(b)** Force applied to one of the C-termini of the chimeric PCDH15 EC1-2-8-10 + CDH23 EC1-2 complex at 1 nm ns^{-1} (average of S14b1-3, teal) and 0.1 nm ns^{-1} (S14a, olive). A 1 ns running average is shown in each case. The gray bars represent the standard deviation of the force for 4 and 3 independent simulations for the PCDH15 EC8-10 and the chimeric PCDH15 EC1-2-8-10 + CDH23 EC1-2 complex at 1 nm ns^{-1}, respectively. Horizontal colored dashed lines highlight maximum force peak for simulations at 1 and 0.1 nm ns^{-1}.The unbending phase (phase I) is clearly observable for both systems. Unbinding of PCDH15 from CDH23 in the chimeric complex (phase II in b) requires less force than unfolding of EC10 (phase II in a).

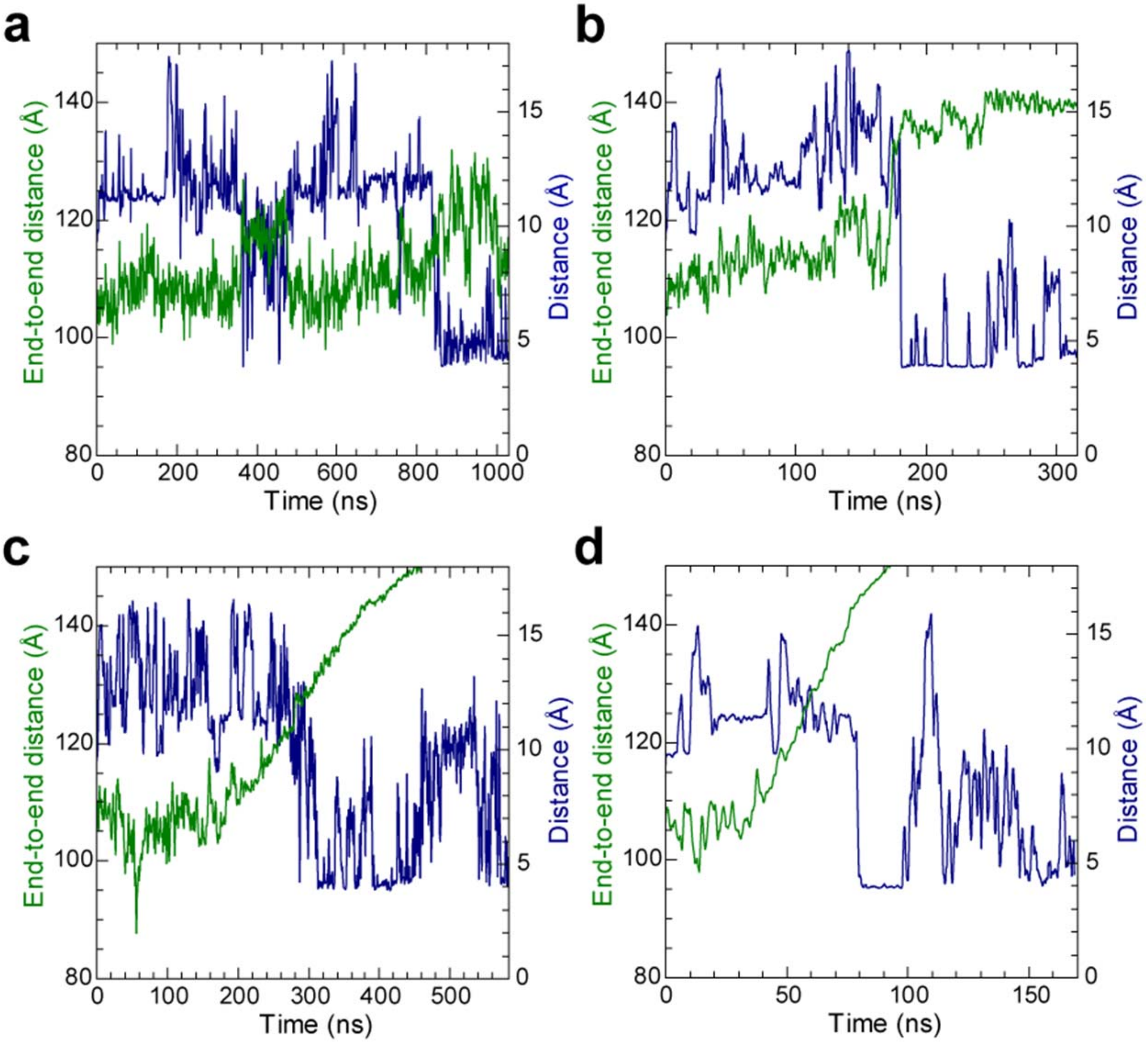

Supplementary Figure 13. Interactions between Glu 1010 and Arg 1013. End-to-end distance (Ser 975:C_α – Glu 1116: C_α, dark green) and distance between Glu 1010:C_γ and Arg 1013:C_ζ (blue) for (**a**) constant-force stretching at 10 pN (S3a; 5 ns running average) and (**b**) 25 pN (S3d; 1 ns running average) as well as constant-velocity stretching at (**c**) 0.02 nm ns^{-1} (S5e; 5 ns running average) and (**d**) 0.1 nm ns^{-1} (S5b; 1 ns running average).

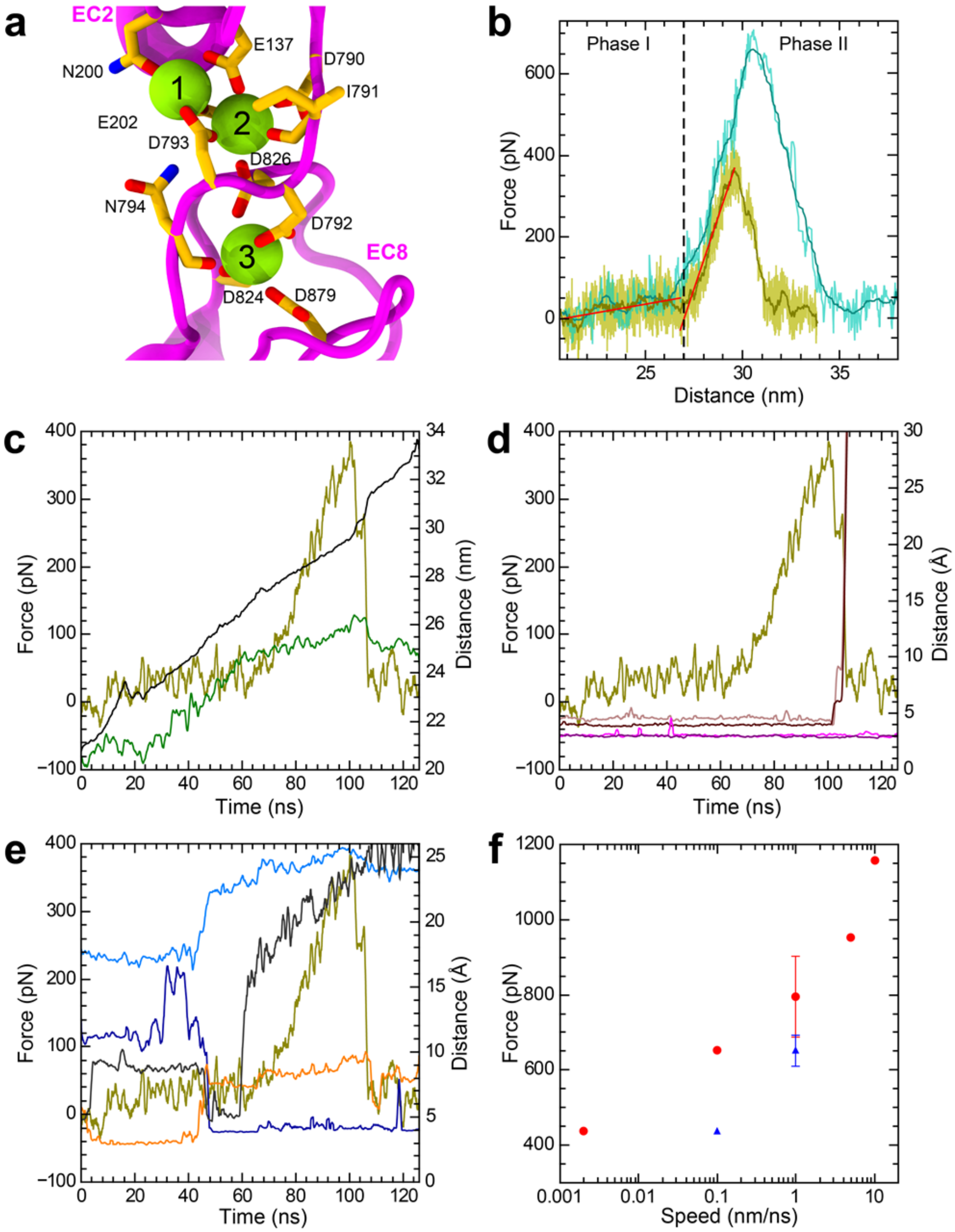

Supplementary Figure 14. Molecular correlates of forced unbending and unbinding of the chimeric PCDH15 EC1-2-8-10 + CDH23 EC1-2 complex. (**a**) Detail of the calcium-binding sites at the chimeric PCDH15 EC2-8 linker. (**b**) Force applied to one of the C-termini vs. end-to-end distance of the chimeric complex (CDH23 Glu 208:C_α – PCDH15 Glu 1116: C_α) for constant-velocity stretching at 1 nm ns^{-1} (simulation S14b1, 1 ns running average, teal) and 0.1 nm ns^{-1} (S14a, 1 ns running average, olive). Red lines show linear fittings for the estimation of spring constants in phases I and II. Lighter colored lines correspond to 50 ps running averages. (**c**) Force applied to one of the C-termini for simulation S14a (olive, left scale). End-to-end distances for the chimeric complex (CDH23 Glu 208:C_α – PCDH15 Glu 1116:C_α) and PCDH15 EC1-2-8-10 (Gln 1:C_α – Glu 1116: C_α) are also shown in black and green, respectively (right scale). (**d-e**) Distances between key residues were monitored throughout an SMD simulation performed at 0.1 nm ns^{-1} (S14a): (**d**) PCDH15 Arg 113:C_ζ – CDH23 Glu 77:C_δ (light brown), PCDH15 Arg 84:C_ζ – CDH23 Asn 96:C_γ (dark brown), Arg 1013:O – Ala 1040:N (magenta), Arg 1013:N – Ala 1040:O (purple). These show that unbinding occurs without unfolding of EC10. (**e**) Distances for: Val 1005:C_α – Arg 1013:C_α (light blue), Gly 1009:O – Ile 1042:N (orange), Glu 1010:C_γ – Arg 1013:C_ζ (blue) and Leu 1004:C_δ – Ala 1096:C_β (dark gray). These show that unbending of EC9-10 occurs before unbinding. A 1 ns running average is shown in all cases. (**f**) Maximum force-peak values vs. stretching speed for simulations of PCDH15 EC8-10 (S5a-e; ●) and the chimeric complex (S14a-b; ▲). Red and blue bars show the standard deviation for independent simulations for the PCDH15 EC8-10 (n = 4) and the chimeric PCDH15 EC1-2-8-10 + CDH23 EC1-2 complex (n = 3) at 1 nm ns^{-1}. Overall, monitored forces and relevant distances indicate that separation of the complex occurred without unfolding.

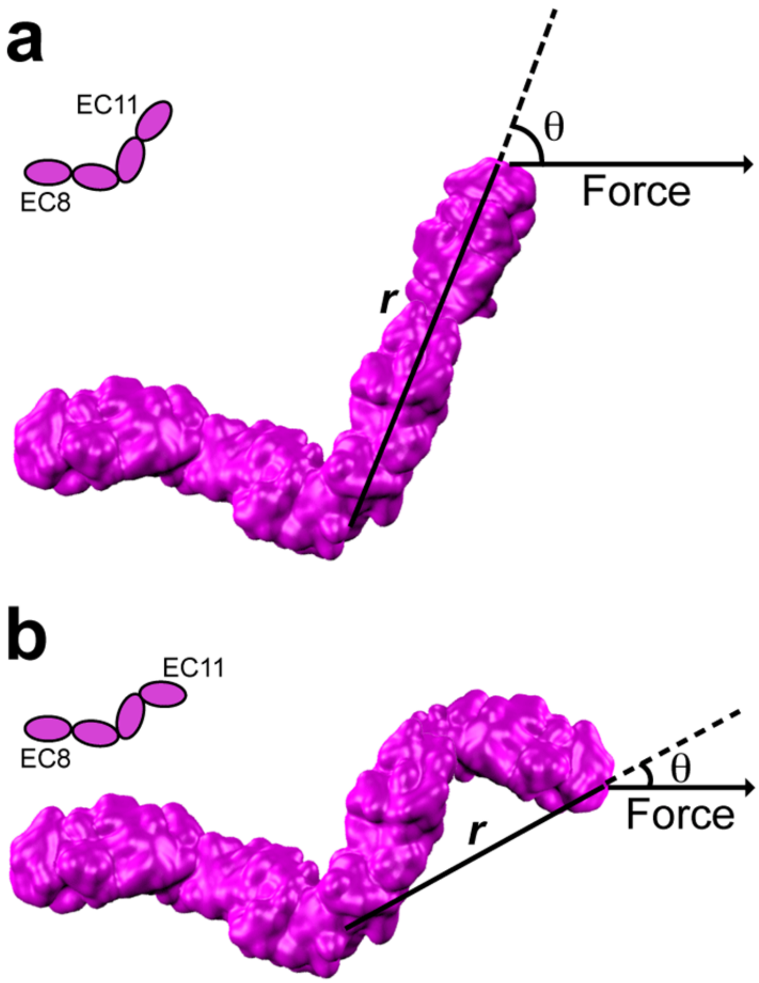

Supplementary Figure 15. Hypothetical models of torque applied to the EC9-10 linker in tip link fragments that include EC11. The arrangement of EC repeats that follow EC10 will alter the torque $\vec{\tau}$ applied to the EC9-10 linker by a force $\vec{F}$, as determined using:

$$|\vec{\tau}| = |\vec{r} \times \vec{F}| = rF\sin\theta,$$

where $\vec{r}$ is the length of the "lever arm" defined as the distance between the EC9-10 linker and the C-terminus of EC11, and θ is the angle between the direction in which the force is applied and the direction along the lever arm. Two hypothetical models are presented, as the placement of EC11 is uncertain. The human EC10-11 linker lacks one of the residues needed to coordinate a calcium ion (DXNDN is replaced by DXN**N**H), therefore the EC10-11 linker could be **(a)** straight or **(b)** kinked (~90°) as illustrated. In the first model, less force would be required to unbend EC9-10. In the second, more force is required to unbend it. More complete structural models are required to elucidate which scenario happens *in vivo*.

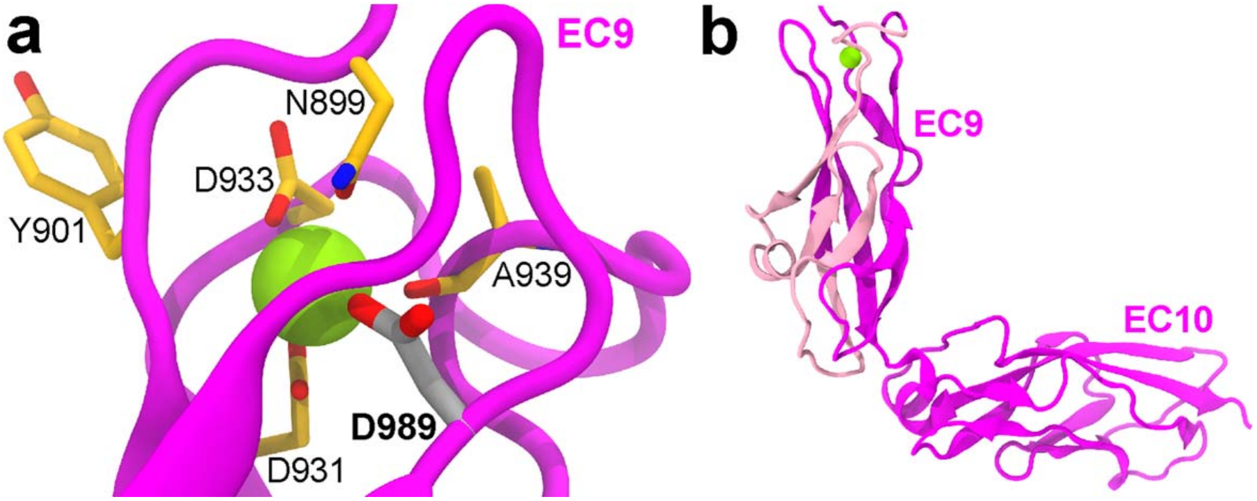

Supplementary Figure 16. Deafness-related variations in PCDH15 EC8-10. (**a**) Detail of the PCDH15 EC9 calcium-binding domain highlighting residue Asp 989 (gray). The mutation D989G is associated to non-syndromic hereditary deafness[5]. (**b**) PCDH15 EC9-10 shown as a magenta ribbon highlighting in pink a large in-frame deletion associated with hereditary deafness[6].

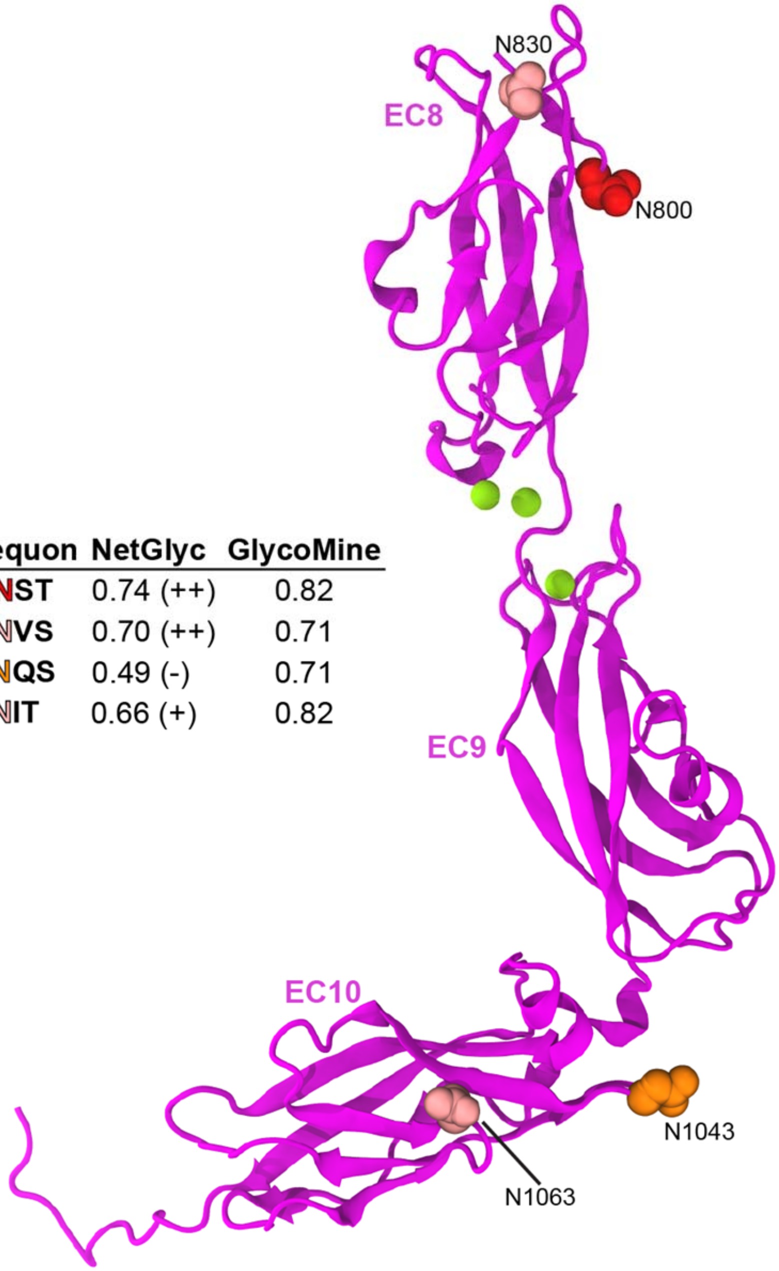

Residue	Sequon	NetGlyc	GlycoMine
Asn 800	NST	0.74 (++)	0.82
Asn 830	NVS	0.70 (++)	0.71
Asn 1043	NQS	0.49 (-)	0.71
Asn 1063	NIT	0.66 (+)	0.82

Supplementary Figure 17. Potential N-glycosylation sites in PCDH15 EC8-10. Structure of PCDH15 EC8-10 showing the position of Asn residues that are part of a “N-X-S/T” motif (where X is any residue except Proline). Residues colored in red are potentially N-glycosylated according to NetNGlyc[7] and GlycoMine[8].

Supplementary Table 1. Comparison of distances between calcium ions at position 2-3 for available cadherin structures

Protein	PDB id	Distance (Å)
PCDH15 EC 8-9	4XHZ	8.48 (8.1 ± 0.2)
PCDH15 EC1-2	4APX_B	6.64
	4AQ8_C	6.72
	4AQ8_D	6.72
CDH23 EC1-2	2WHV	6.81
	4APX_A	6.78
	4AQ8_A	6.73
	4AQ8_B	6.73
	3MVS	6.8
NCAD EC1-2	3Q2W	6.64
NCAD EC2-3		6.67
NCAD EC3-4		6.85
NCAD EC4-5		6.8
CCAD EC1-2	1L3W	6.71
CCAD EC2-3		6.44
CCAD EC3-4		7.25
CCAD EC4-5		7.93
ECAD EC1-2	3QV2	6.7
ECAD EC2-3		7.17
ECAD EC3-4		7.37
ECAD EC4-5		7.17
DNCAD EC 1-2	3UBH	6.91
DNCAD EC 3-4		6.73
VECAD EC1-2	3PPE_A	6.78
PCDHγC3 EC1-2	4ZI8	6.61
PCDHγC3 EC2-3		6.67
PCDHγA1 EC1-2	4ZI9_A	6.71
PCDHγA1 EC2-3		6.7
PCDHβ1 EC1-2	4ZPL_A	7.27
PCDHβ1 EC2-3		7.2
PCDHαC2 EC1-2	4ZPM_A	6.5
PCDHαC2 EC2-3		6.58
PCDHγC5 EC1-2	4ZPN_A	6.65
PCDHγC5 EC2-3		7.17
PCDHγC5 EC1-2	4ZPO_A	6.78
PCDHγC5 EC2-3		6.66
PCDHγC5 EC1-2	4ZPP_A	6.72
PCDHγC5 EC2-3		7.04
PCDHγC5 EC1-2	4ZPQ_A	6.6
PCDHγC5 EC2-3		6.79
PCDHγA8 EC1-2	4ZPS_A	6.95
PCDHγA8 EC2-3		7.01

Supplementary Table 2. Summary of MD simulations

Label	PDB	#	t_{sim} (ns)	Type	Ensemble	SMD atoms	Speed or Force	Start	# Atoms	Size (nm^3)
PCDH15 EC8-10 S	4XHZ	S1a	1.21	MinEQ	*NpT**	--	--	--	115625	16x10x7.5
PCDH15 EC8-10 S	4XHZ	S1b	400	EQ	*NpT*	--	--	S1a	115625	16x10x7.5
PCDH15 EC8-10 S	4XHZ	S1c	500	EQ	*NpT¥*	--	--	S1a	115625	16x10x7.5
PCDH15 EC8-10 B	4XHZ	S1d	1.21	MinEQ	*NpT**	--	--	--	204024	28x10x7.5
PCDH15 EC8-10 B	4XHZ	S1e	54.9	EQ	*NpT*	--	--	S1d	204024	28x10x7.5
PCDH15 EC8-10 B	4XHZ	S2a	5.0	PCV	*NpT*	COM	2 nm ns^{-1}	S1e	204024	28x10x7.5
PCDH15 EC8-10 B	4XHZ	S2b	50	PCV	*NpT*	COM	0.2 nm ns^{-1}	S1e	204024	28x10x7.5
PCDH15 EC8-10 B	4XHZ	S2c	5.0	PCV	*NpT*	COM	10 nm ns^{-1}	S1e	204024	28x10x7.5
PCDH15 EC8-10 B	4XHZ	S2d	5.0	PCV	*NpT*	COM	20 nm ns^{-1}	S1e	204024	28x10x7.5
PCDH15 EC8-10 B	4XHZ	S3a	1032	PCF	*NpT*	N & C-ter	10 pN	S1e	204024	28x10x7.5
PCDH15 EC8-10 B	4XHZ	S3b	93.9	PCF	*NpT*	N & C-ter	50 pN	S1e	204024	28x10x7.5
PCDH15 EC8-10 B	4XHZ	S3c	56.5	PCF	*NpT*	N & C-ter	100 pN	S1e	204024	28x10x7.5
PCDH15 EC8-10 B	4XHZ	S3d	330.3	PCF	*NpT*	N & C-ter	25 pN	S1e	204024	28x10x7.5
PCDH15 EC8-10 B	4XHZ	S4a	20.0	EQ	*NpT*	--	--	S2a (3.8ns)	204024	28x10x7.5
PCDH15 EC8-10 B	4XHZ	S4b	20.0	EQ	*NpT*	--	--	S2a (4.0 ns)	204024	28x10x7.5
PCDH15 EC8-10 B	4XHZ	S4c	46.4	EQ	*NpT*	--	--	S2a (4.3 ns)	204024	28x10x7.5
PCDH15 EC8-10 B	4XHZ	S4d	20.0	EQ	*NpT*	--	--	S2a (5.0ns)	204024	28x10x7.5
PCDH15 EC8-10 B	4XHZ	S5a1	30.5	PCV	*NpT*	N & C-ter	1 nm ns^{-1}	S1e	204024	28x10x7.5
PCDH15 EC8-10 B	4XHZ	S5a2	28.7	PCV	*NpT*	N & C-ter	1 nm ns^{-1}	S1e	204024	28x10x7.5
PCDH15 EC8-10 B	4XHZ	S5a3	33.4	PCV	*NpT*	N & C-ter	1 nm ns^{-1}	S1e	204024	28x10x7.5
PCDH15 EC8-10 B	4XHZ	S5a4	30.8	PCV	*NpT*	N & C-ter	1 nm ns^{-1}	S1e	204024	28x10x7.5
PCDH15 EC8-10 B	4XHZ	S5b	168.7	PCV	*NpT*	N & C-ter	0.1 nm ns^{-1}	S1e	204024	28x10x7.5
PCDH15 EC8-10 B	4XHZ	S5c	5.0	PCV	*NpT*	N & C-ter	5 nm ns^{-1}	S1e	204024	28x10x7.5
PCDH15 EC8-10 B	4XHZ	S5d	5.0	PCV	*NpT*	N & C-ter	10 nm ns^{-1}	S1e	204024	28x10x7.5
PCDH15 EC8-10 B	4XHZ	S5e	583.1	PCV	*NpT*	N & C-ter	0.02 nm ns^{-1}	S1e	204024	28x10x7.5
PCDH15 EC8-10 B	4XHZ	S6a	19.2	PCV	*NpT*	COM	1 nm ns^{-1}	S1e	204024	28x10x7.5
PCDH15 EC8-10 B	4XHZ	S6b	11.9	PCV	*NpT*	COM	5 nm ns^{-1}	S1e	204024	28x10x7.5
PCDH15 EC8-10 B	4XHZ	S6c	1.7	PCV	*NpT*	COM	10 nm ns^{-1}	S1e	204024	28x10x7.5
PCDH15 EC8-10 B	4XHZ	S7a	75.7	EQ	*NpT*	--	--	S3c (3ns)	204024	28x10x7.5
PCDH15 EC8-10 B	4XHZ	S7b	83.2	EQ	*NpT*	--	--	S3c (7.5ns)	204024	28x10x7.5
PCDH15 EC8-10 B	4XHZ	S7c	101.6	EQ	*NpT*	--	--	S3c (10ns)	204024	28x10x7.5
PCDH15 EC8-10 B	4XHZ	S7d	101.4	EQ	*NpT*	--	---	S3c (26ns)	204024	28x10x7.5
PCDH15 EC8-10 B	4XHZ	S8a	12.6	PCV	*NVE*	N & C-ter	1 nm ns^{-1}	S1e	204024	28x10x7.5
PCDH15 EC8-10 B	4XHZ	S8b	10.0	PCV	*NVE*	N & C-ter	5 nm ns^{-1}	S1e	204024	28x10x7.5
PCDH15 EC8-10 B	4XHZ	S8c	5.0	PCV	*NVE*	N & C-ter	10 nm ns^{-1}	S1e	204024	28x10x7.5
PCDH15 EC8-10 B	4XHZ	S9a	90.5	EQ	*NpT*	--	--	S5b (60ns)	204024	28x10x7.5
PCDH15 EC8-10 B	4XHZ	S9b	88.7	EQ	*NpT*	--	--	S5b (67ns)	204024	28x10x7.5
PCDH15 EC8-10 B	4XHZ	S9c	115.9	EQ	*NpT*	--	--	S5b (70ns)	204024	28x10x7.5
PCDH15 EC8-10 B	4XHZ	S9d	108.8	EQ	*NpT*	--	--	S5b (75ns)	204024	28x10x7.5
PCDH15 EC8-10 B	4XHZ	S9e	110.2	EQ	*NpT*	--	--	S5b (76ns)	204024	28x10x7.5
PCDH15 EC8-10 B	4XHZ	S9f	78.4	EQ	*NpT*	--	--	S5b (81ns)	204024	28x10x7.5
PCDH15 EC8-10 B	4XHZ	S9g	78.0	EQ	*NpT*	--	--	S5b (101ns)	204024	28x10x7.5
		**Total**	**4619.4**							
DNcad EC2-3	3UBG	S10a	1.21	MinEQ	*NpT**	--	--	--	134046	22x7.1x8.9

DNcad EC2-3	3UBG	S10b	50	EQ	*NpT*	--	--	S10a	134046	22x7.1x8.9
DNcad EC2-3	3UBG	S11a	113.7	PCF	*NpT*	N & C-ter	10 pN	S10b	134046	22x7.1x8.9
DNcad EC2-3	3UBG	S11b	83.5	PCF	*NpT*	N & C-ter	50 pN	S10b	134046	22x7.1x8.9
DNcad EC2-3	3UBG	S11c	86.5	PCF	*NpT*	N & C-ter	100 pN	S10b	134046	22x7.1x8.9
DNcad EC2-3	3UBG	S12a	55.2	PCV	*NpT*	N & C-ter	1 nm ns^{-1}	S10b	134046	22x7.1x8.9
DNcad EC2-3	3UBG	S12b	96	PCV	*NpT*	N & C-ter	0.1 nm ns^{-1}	S10b	134046	22x7.1x8.9
		**Total**	**486.1**							
Chimeric Complex		S13a1	1.21	MinEQ	*NpT**	--	--	--	209952	44x6.9x7.3
Chimeric Complex		S13a2	1.21	MinEQ	*NpT**	--	--	--	209942	44x6.9x7.3
Chimeric Complex		S13b	30	EQ	*NpT*			S13a1	209952	44x6.9x7.3
Chimeric Complex		S13c	28.8	EQ	*NpT*			S13a2	209942	44x6.9x7.3
Chimeric Complex		S14a	126	PCV	*NpT*	C & C-ter	0.1 nm ns^{-1}	S13b	209952	44x6.9x7.3
Chimeric Complex		S14b1	17.4	PCV	*NpT*	C & C-ter	1 nm ns^{-1}	S13b	209952	44x6.9x7.3
Chimeric Complex		S14b2	17.7	PCV	*NpT*	C & C-ter	1 nm ns^{-1}	S13c (28.8ns)	209942	44x6.9x7.3
Chimeric Complex		S14b3	16.4	PCV	*NpT*	C & C-ter	1 nm ns^{-1}	S13c (13.8ns)	209942	44x6.9x7.3
		**Total**	**238.7**							
PCDH15 EC9-10$_{S5b\text{-}75ns}$		S15a	10	EQ	*NpT*	--	--	S5b (75ns)	105545	13x9.1x9.2
PCDH15 EC9-10$_{S5b\text{-}75ns}$		S15b	2202	EQ§	*NpT*	--	--	S15a	105545	13x9.1x9.2
PCDH15 EC9-10$_{S5b\text{-}76ns}$		S16a	10	EQ	*NpT*	--	--	S5b (76ns)	98124	13x9.2x8.8
PCDH15 EC9-10$_{S5b\text{-}76ns}$		S16b	7341	EQ§	*NpT*	--	--	S16a	98124	13x9.2x8.8
		**Total**	**9563**							
		**Total**	**14907.3**							

Summary of all MD simulations**.** Labels and annotations as in Table 2. Asterisk (*) denotes simulations that consisted of 5000 steps of minimization, 200 ps of dynamics with the backbone of the protein restrained (k_{bbr} = 1 kcal mol^{-1} Å^{-2}), and 1 ns of free dynamics in the *NpT* ensemble (γ = 1 ps^{-1}). ¥ Denotes an equilibrium simulation with restraints applied (k_r = 1 kcal mol^{-1} Å^{-2}) to C_α atoms of residues 797, 833, 876 and 888 of EC8. COM indicates simulations in which force was applied to the center of mass of multiple atoms: C_α:795-798; 881-884; 825-829 from EC8 and C_α:1024-1028;1074-1080;1113-1116. § Denotes equilibrium simulations performed in Anton (see Methods).

Supplementary Table 3. SAXS data collection and scattering-derived parameters

**Data-collection parameters**	
Instrument	MAR-165
Beam geometry	1.5 meters distance
Wavelength (Å)	1.0332
q range $Å^{-1}$	0.01 - 0.32
Exposure times (sec)	0.5, 1, 2, 5
Concentration range (mg/ml)	1-2
Temperature (K)	293.15
**Structural parameters** *	
R_g (Å) [from P(r)]	40.61 ± 0.291
R_g (Å) [from Guinier]	37.1 ± 1.1
D_{max} (Å)	130 ± 10
**Molecular mass determination** *§	
V_c [Guinier]	414.38
V_c [P(r)]	432.61
Molecular mass [Guinier]	37.6 KDa
Molecular mass [P(r)]	37.4 KDa
Calculated mass from sequence	38.9 KDa
**Software employed**	
Primary data reduction	Beamline specific software
Data processing	PRIMUS, GNOM
Ab initio analysis	DAMMIF
Validation and averaging	DAMAVER
Computation of model intensities	FoXS

* Reported for 1 mg mL^{-1} measurement. § Molecular mass was estimated using the method described in [9] using R_g obtained from the Guinier analysis and from P(r).

SUPPLEMENTARY DISCUSSION

Crystallographic Contacts

Parallel dimerization of PCDH15 and CDH23 is supported by biochemical data obtained using full-length extracellular domains[10], yet CDH23 EC1-2[11,12], PCDH15 EC8-10, and mmPcdh15 EC9-10 are monomeric in solution. Crystallographic contacts for the EC8-10 and EC9-10 structures seem to support the monomeric states of these fragments. The two structures show high similarity in their overall fold and orientation of EC9 and EC10 with an RMSD-C_α of ~0.5 Å (Supplementary Fig. 7a-c). Three crystal contacts were observed in the EC8-10 structure (Supplementary Fig. 7d,g,j), while four were observed for the EC9-10 structure (Supplementary Fig. 7e,h,k,l).

Two interfaces found in both structures are similar to each other. The first one corresponds to a pseudo-parallel dimer conformation in which the EC9-10 bent linkers interact with both the N- and C-termini of the protomers pointing away from each other (Supplementary Fig. 7g,h). Residues from loops AB and BE from EC9 (near the EC9-10 linker), residues from the EC9-10 3_{10} helix, and residues from the BC loop of EC10 interact in a symmetrical fashion with interfacial areas of 509 $Å^2$ for EC8-10 and 460 $Å^2$ for EC9-10 (Supplementary Fig. 7g,h). Superposition of both dimer pairs shows a remarkable similarity with an RMSD-C_α of ~0.5 Å (Supplementary Fig. 7i). Analysis of this interface with *NOX-class*[13] indicates that it most likely corresponds to a crystal packing contact. The second interface corresponds to an “anti-parallel dimer” formed mostly by EC10 with interfacial areas of 1471 $Å^2$ for EC8-10 and 1043 $Å^2$ for EC9-10 (Supplementary Fig. 7d,e). Superposition of both dimer pairs shows that despite having similar orientations, they do not align well (Supplementary Fig. 7f).

Other crystal contacts are shown in Supplementary Fig. 7j,k,l. Because none of the crystal contacts seem to represent a physiologically relevant interface, parallel dimerization of PCDH15 might be mediated by other EC repeats, or by multiple small non-specific contacts as observed by negative staining transmission electron microscopy[10].

Deafness-related variations in PCDH15 EC8-10.

Two deafness-related variations can be mapped to PCDH15 EC8-10. The mutation D989G associated to non-syndromic hereditary deafness[5] (Supplementary Fig. 16a) affects the XDX^{TOP} motif of EC9. This mutation will likely impair calcium binding and perhaps folding[1,14]. The second variation corresponds to a large in-frame deletion that removes strands C’, D’, E’ and half of F’ in EC9. Several lines of evidence indicate that unstable tip links are formed in the presence of this deletion (Supplementary Fig. 16b)[6,15]. However, the effects of this deletion on hair-cell gating compliance, adaptation, and tip-link mechanics are unknown.

Predicted glycosylation sites in PCDH15 EC8-10.

N-linked glycosylation may affect the adhesive properties of cadherins[16]. Prediction of potential N-glycosylation sites on PCDH15 EC8-10 was carried out to search for sites that may affect the conformation and elastic response of the EC9-10 linker. Four sites that match the Asn–X–Ser/Thr motif (sequon) were found on the PCDH15 EC8-10 sequence (Supplementary Fig. 17). Prediction servers NetGlyc[7] and GlycoMine[8] identify only one site with high confidence (Asn 800 in EC8). The only potential N-glycosylation site found near the EC9-10 interface corresponds to Asn 1043, part of the EC10 support loop. This residue is located in the middle of the 1040-1046 loop with its side chain pointing towards the solvent. It is unlikely that N-glycosylation at this site will interfere in the EC9-10 interface. However, the presence or absence of a sugar in this position may modulate its elastic response.

Interatomic distances used as markers for unfolding, unbending, and unbinding.

Trajectories of SMD simulations involving PCDH15 EC8-10 and the chimeric complex of PCDH15 EC1-2-8-9-10 + CDH23 EC1-2 were inspected to find molecular events that correlate with unbending of PCDH15 EC9-10, unfolding of PCDH15 EC10 and unbinding of PCDH15 EC1-2 from CDH23 EC1-2. As shown in Figs. 6b&c, 7c-f and Supplementary Fig. 14c-e, unbending of PCDH15 EC9-10 correlates with the rupture of backbone hydrogen bonds formed between His 1007:O and Glu 1010:N (both part of the EC9-10 3_{10} helix), as well as Gly 1009:O (EC9-10 linker) and Ile 1042:N (EC10 support loop). The beginning of PCDH15 EC10 unfolding observed after unbending in constant-velocity SMD simulations correlates with the rupture of a pair of backbone hydrogen bonds formed by Arg 1013 (EC9-10 linker) and Ala 1040 (EC10 support loop). The rupture of these hydrogen bonds coincides with the first force peak observed in the constant-velocity simulations at 1, 0.1 and 0.02 nm ns^{-1}. A second, larger force peak observed in these simulations correlates with the rupture of the backbone hydrogen bonds Tyr 1019:N – Lys 1108:O and Tyr 1019:O – Tyr 1110:N that leads to "unzipping" of β strands F'' and G'' in EC10. To monitor unbinding of PCDH15 EC1-2 from CDH23 EC1-2 we used interatomic distances between sidechains of PCDH15 Arg 113 and CDH23 Glu 77, as well as between PCDH15 Arg 84 and CDH23 Asn 96[1].

Simulation timescales and physiological stimuli.

Our SMD simulations are limited to relatively short timescales, with constant-force simulations reaching ~1 μs and constant-velocity simulations using stretching speeds ≥0.02 nm ns^{-1}. How do they compare to physiologically relevant timescales and speeds? While many biological processes occur in timescales of milliseconds, inner-ear hair cells must handle fast stimuli associated with high-frequency sound (up to ~20 KHz for human auditory perception). Physiologically relevant timescales for hair cell mechanotransduction can be as short as 10 to 50 μs[17], with basilar membrane speeds reaching ~0.01 m s^{-1} for a stimulus at 7 KHz and 95 dB in the chinchilla's cochlea[18]. The extension of tip links x_t can be written as:

$$x_t = \gamma X_b + x_{to},$$

where X_b is the position of the hair bundle, x_{to} is the resting length of the tip link, and γ is a geometrical gain determined by the arrangement of tip links within a bundle[19]. For a hair-cell bundle moving at speeds similar to those experienced by the basilar membrane at 7 KHz, and assuming $\gamma \approx 0.14$[19], tip links will be stretched at $v_t \approx \gamma \times 0.01$ m s^{-1} = 0.0014 m s^{-1}. Thus, the timescales and stretching speeds used in our simulations (0.02 m s^{-1}) are within one order of magnitude of what real tip links could normally experience *in vivo*, and estimates of unfolding strength and tip link stiffness must be carefully interpreted, as slower pulling speeds will result in lower predicted values[20-23]. It is possible that our simulations only predict well the mechanical response of tip links to loud auditory insults (>95 dB at frequencies >7 KHz), and that longer and slower stretching simulations reveal a different mechanical response that better predicts the behavior of PCDH15 EC8-10 and tip-link proteins under normal conditions *in vivo*. However, several experimental studies have confirmed predictions of mechanical properties of proteins for even faster SMD simulations[24-27]. Predictions presented here await further experimental confirmation.

SUPPLEMENTARY REFERENCES

1. Sotomayor, M., Weihofen, W. A., Gaudet, R. & Corey, D. P. Structure of a force-conveying cadherin bond essential for inner-ear mechanotransduction. *Nature* **492,** 128–32 (2012).
2. Ciatto, C. *et al.* T-cadherin structures reveal a novel adhesive binding mechanism. *Nat. Struct. Mol. Biol.* **17,** 339–47 (2010).
3. Jin, X. *et al.* Crystal structures of Drosophila N-cadherin ectodomain regions reveal a widely used class of Ca^{2}+-free interdomain linkers. *Proc. Natl. Acad. Sci. U. S. A.* **109,** E127–34 (2012).
4. Rambo, R. P. & Tainer, J. A. Characterizing flexible and intrinsically unstructured biological macromolecules by SAS using the Porod-Debye law. *Biopolymers* **95,** 559–71 (2011).
5. Chen, D. *et al.* Mutation in PCDH15 may modify the phenotypic expression of the 7511T>C mutation in MT-TS1 in a Chinese Han family with maternally inherited nonsyndromic hearing loss. *Int. J. Pediatr. Otorhinolaryngol.* **79,** 1654–1657 (2015).
6. Alagramam, K. N. *et al.* Mutations in Protocadherin 15 and Cadherin 23 Affect Tip Links and Mechanotransduction in Mammalian Sensory Hair Cells. *PLoS One* **6,** e19183 (2011).
7. Gupta, R. & Brunak, S. Prediction of glycosylation across the human proteome and the correlation to protein function. *Pac. Symp. Biocomput.* **322,** 310–322 (2002).
8. Li, F. *et al.* GlycoMine: a machine learning-based approach for predicting N-, C- and O-linked glycosylation in the human proteome. *Bioinformatics* **31,** 1411–9 (2015).
9. Rambo, R. P. & Tainer, J. A. Accurate assessment of mass, models and resolution by small-angle scattering. *Nature* **496,** 477–81 (2013).
10. Kazmierczak, P. *et al.* Cadherin 23 and protocadherin 15 interact to form tip-link filaments in sensory hair cells. *Nature* **449,** 87–91 (2007).
11. Elledge, H. M. *et al.* Structure of the N terminus of cadherin 23 reveals a new adhesion mechanism for a subset of cadherin superfamily members. *Proc. Natl. Acad. Sci. U. S. A.* **107,** 10708–12 (2010).
12. Sotomayor, M., Weihofen, W. a., Gaudet, R. & Corey, D. P. Structural Determinants of Cadherin-23 Function in Hearing and Deafness. *Neuron* **66,** 85–100 (2010).
13. Zhu, H., Domingues, F. S., Sommer, I. & Lengauer, T. NOXclass: prediction of protein-protein interaction types. *BMC Bioinformatics* **7,** 27 (2006).
14. Sotomayor, M., Weihofen, W. A., Gaudet, R. & Corey, D. P. Structural Determinants of Cadherin-23 Function in Hearing and Deafness. *Neuron* **66,** 85–100 (2010).
15. Zheng, Q. Y. *et al.* A new spontaneous mutation in the mouse protocadherin 15 gene. *Hear. Res.* **219,** 110–20 (2006).
16. Leckband, D. E. & de Rooij, J. Cadherin adhesion and mechanotransduction. *Annu. Rev. Cell Dev. Biol.* **30,** 291–315 (2014).
17. Corey, D. P. & Hudspeth, A. J. Response latency of vertebrate hair cells. *Biophys. J.* **26,** 499–506 (1979).
18. Robles, L. & Ruggero, M. A. Mechanics of the mammalian cochlea. *Physiol. Rev.* **81,** 1305–52 (2001).
19. Howard, J. & Hudspeth, a J. Compliance of the hair bundle associated with gating of mechanoelectrical transduction channels in the bullfrog's saccular hair cell. *Neuron* **1,** 189–199 (1988).
20. Evans, E. & Ritchie, K. Dynamic strength of molecular adhesion bonds. *Biophys. J.* **72,** 1541–1555 (1997).
21. Evans, E. A. & Calderwood, D. A. Forces and bond dynamics in cell adhesion. *Science* **316,** 1148–53 (2007).
22. Dudko, O. K., Hummer, G. & Szabo, A. Theory, analysis, and interpretation of single-molecule force spectroscopy experiments. *Proc. Natl. Acad. Sci. U. S. A.* **105,** 15755–60 (2008).
23. Dudko, O. K. Decoding the mechanical fingerprints of biomolecules. *Q. Rev. Biophys.* 1–14 (2015). doi:10.1017/S0033583515000220
24. Lee, G. *et al.* Nanospring behaviour of ankyrin repeats. *Nature* **440,** 246–249 (2006).

25. Sotomayor, M. & Schulten, K. Single-molecule experiments in vitro and in silico. *Science* **316,** 1144–8 (2007).
26. Oroz, J. *et al.* Nanomechanics of the cadherin ectodomain: 'canalization' by Ca2+ binding results in a new mechanical element. *J. Biol. Chem.* **286,** 9405–18 (2011).
27. Rico, F., Gonzalez, L., Casuso, I., Puig-Vidal, M. & Scheuring, S. High-speed force spectroscopy unfolds titin at the velocity of molecular dynamics simulations. *Science* **342,** 741–3 (2013).
